# The Epigenomics of Migraine

**DOI:** 10.1007/s11916-026-01574-5

**Published:** 2026-10-29

**Authors:** Yi-Chia Liaw

**Affiliations:** 1 Department of Internal Medicine, National Yang-Ming Chiao-Tung University Hospital, Yi-Lan, Taiwan; 2 Institute of Clinical Medicine, School of Medicine, National Yang-Ming Chiao-Tung University, Taipei, Taiwan

## Abstract

**Purpose of Review:**

To review current evidence on epigenetic mechanisms in migraine, offering insights into diagnosis, disease classification, and future therapeutic management.

**Recent Findings:**

Recent research has focused on three primary epigenetic mechanisms: DNA methylation, non-coding RNAs, and histone modifications. Altered methylation patterns have been identified in CALCA and RAMP1, both critical components of the CGRP signaling pathway. Furthermore, recent research on non-coding RNA data has enabled the development of composite risk scores to distinguish endophenotypes and support clinical diagnosis. Lastly, the therapeutic efficacy of valproic acid may be mediated via its endogenous histone deacetylase-inhibiting effect and chromatin remodeling.

**Summary:**

As our understanding of epigenetic modulation in migraine expands, these molecular insights can be integrated with genomic profiles to advance precision medicine, adding value to the interaction between genetic and environmental factors.

## Introduction

Migraine is a complex but prevalent neurovascular disorder that can cause tremendous daily disability. Consequently, extensive research aimed at establishing classification systems, refining diagnostic criteria, developing targeted therapeutic strategies, as well as monitoring treatment efficacy has gained significant attention. With recent advancements in molecular biology and computational techniques, research has increasingly shifted toward personalized and precision medicine. A prominent approach has been genome-wide association studies (GWAS), which have revolutionized our understanding of migraine [1]. However, using merely single-layered genetic analyses fails to capture biological functional hierarchy and is insufficient to bridge the gap from genomic risk loci to functional proteins, let alone facilitate clinical translation [2]. Moreover, since the heritability for migraine is between 30 and 60% as published in previous research, the interaction of environmental factors should be taken into account [3]. To overcome these limitations, multi-layered methodologies have emerged. In particular, multi-omics approaches, which integrate blood-based and imaging-based biomarkers, have gained substantial advances. Among these studies [2, 4, 5], genetics, transcriptomics, proteomics, and metabolomics were broadly addressed. By interacting with environmental influences, epigenetic mechanisms also help explain why individuals with similar DNA sequences can exhibit different clinical phenotypes. Since these epigenetic modifications remain largely reversible, they offer promising therapeutic approaches through modulators, as well as crucial insights into disease pathogenesis, diagnostic biomarkers, and risk stratification for migraine management [6]. Thus, in this review, we highlight the recent advances of epigenomics in migraine and discuss how this evidence can change personalized care in migraine management.

## Methods

This narrative review synthesizes current evidence across three key domains of epigenomics in migraine: DNA methylation, non-coding RNAs as well as histone modifications. No prior protocol registration (e.g., PROSPERO) was performed for this study; rather, a structured literature search strategy was applied to conduct a comprehensive narrative overview. A literature search was performed through the PubMed database up to August 1st, 2026. The search incorporated Medical Subject Headings (MeSH) and free-text terms, including: (Migraine) AND (epigenomics), (Migraine) AND (DNA methylation), (Migraine) AND (histone modifications), and (Migraine) AND (non-coding RNA). No restrictions were placed on publication date. Eligibility of the articles was limited to peer-reviewed articles with available full text or a clear abstract published in the English language. All retrieved citations were screened and evaluated for inclusion or exclusion by the author (Y.-C. L.).

**Fig. 1:**
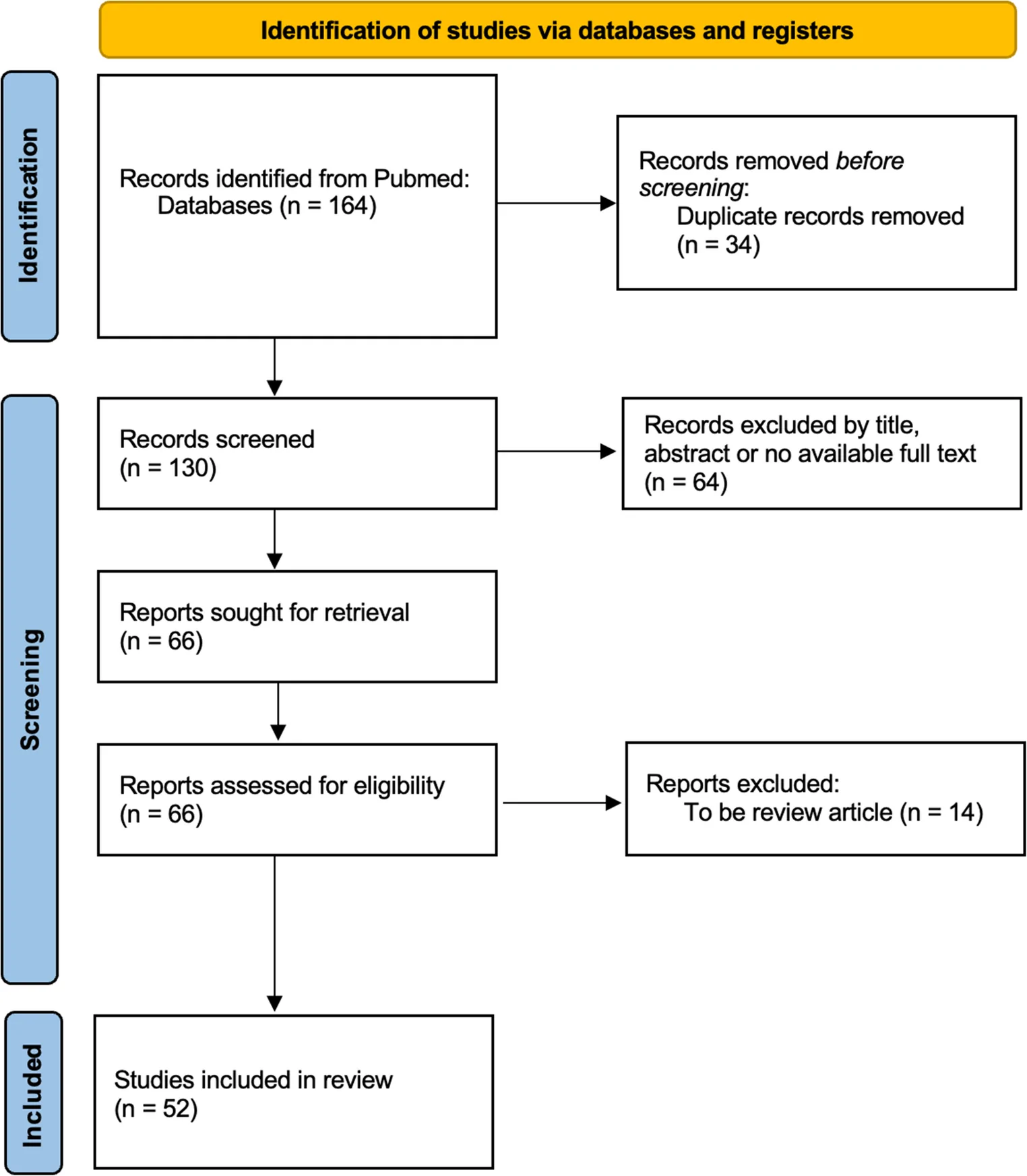
The PRISMA flowchart of this review article

The selection of the articles was demonstrated in Fig. 1. Out of 164 records retrieved initially, 34 duplicates were removed, leaving 130 articles for initial evaluation. Based on the screening results of titles, abstracts, and the available full texts, 64 articles were excluded since the contents did not match the requirements for this review. Among them, 14 articles were review articles and were also removed from the study. Ultimately, 52 articles met the eligibility criteria for review.

As instructed by this journal’s artificial intelligence (AI) usage policy, the author discloses that Gemini 3.7 Flash was used during the editing process to refine and polish the language. The AI tool did not participate in literature searching, file downloading, content screening, or creating interpretations or personal insights. The author retains full responsibility for the final content and its accuracy. The author also declared no financial funding of this review article. All reported studies/experiments with human or animal subjects performed by the authors have been previously published and complied with all applicable ethical standards (including the Helsinki declaration and its amendments, institutional/national research committee standards, and international/national/institutional guidelines).

## Results

A trigeminal ganglia cell atlas of human and mouse provides intriguing evidence for the role of the epigenome in migraine pathophysiology [7]. The atlas revealed that more than 35 of 123 migraine risk single nucleotide polymorphisms (SNPs) found in a previous genome-wide association study (GWAS) [1] align with accessible chromatin peaks, and this overlap suggests that numerous migraine-associated SNP variants may influence disease susceptibility via epigenomic interactions. Moreover, with the integration of multi-omics studies, two key effectors involved in synaptic function were also proposed to act as regulators of migraine pathophysiology [8]. In a review article conducted by Fila et al., the authors have shown that the *TRPA1* gene, one of the important perception receptors, was regulated via multiple epigenetic pathways, including DNA methylation, non-coding RNAs (ncRNAs), and histone modifications [9]. These results together emphasized the important role of epigenetic modulation in migraine.

## DNA Methylation

Dysregulated DNA methylation across migraine-associated target genes represents an important epigenetic mechanism underlying its pathophysiology. In a study enrolling 22 episodic migraine patients and 20 healthy controls, targeted methylation profiling revealed significant hypomethylation at two CpG sites within the CALCA gene, the gene that encodes CGRP. Furthermore, methylation levels at these specific CpG loci correlated significantly with clinical endophenotypes, particularly nausea and vomiting, which again emphasize its critical role in migraine [10]. A study also demonstrated that the regulation of the CALCA gene in different cell types is highly regulated by DNA methylation [11]. The analysis of RAMP1, a subunit of the calcitonin gene-related peptide (CGRP) receptor, revealed a non-significant reduction in overall methylation among migraine patients; nevertheless, the site-specific evaluation demonstrated significantly lower methylation across several CpG units in female patients when compared to the healthy controls [12]. Conversely, RAMP1 promoter hypermethylation among women was reported in another study [13]. These discrepancies in RAMP1 promoter methylation patterns may stem from several factors. First, the cohorts differed in ethnic background (East Asian vs. European). Second, while the former study enrolled both male and female patients, the latter included exclusively female participants, potentially demonstrating sex-specific epigenetic variations. Finally, the interrogated CpG loci were situated in opposite orientations relative to the transcription start site (TSS), which may exert distinct biological and transcriptional effects. Besides the CGRP pathway, an epigenome-wide association study (EWAS) identified 62 independent differentially methylated regions (DMRs), with the most significant region mapping to SLC6A5, while no single-probe associations reached genome-wide significance [14, 15]. Furthermore, the endocannabinoid system (ES) was also studied. A study focusing on the expression of ES receptors CB1 and CB2 has demonstrated that though the genetic expression of both proteins was higher in episodic and chronic migraine patients, the methylation status regarding the promoters of the two genes was indistinguishable when compared to controls, indicating gene expression in this system may be governed through alternative regulatory mechanisms [16]. This evidence together showed that the pain-modulating system was also affected by epigenomic factors.

In a recent case-control study, researchers evaluated the DNA methylation status in patients with migraine without aura, and their findings revealed a significantly higher DNA unmethylation rate in patients compared to healthy controls, of which the result that was also observed in other pro-inflammatory diseases [17, 18]. Furthermore, this hypomethylation status was significantly influenced by the methylenetetrahydrofolate reductase (MTHFR) C677T polymorphism. Based on these findings, researchers have introduced the concept of an “epigenetic diet”, attempting to modulate DNA methylation patterns via modulating mono-carbonic transfer system; however, clinical evidence supporting nutritional supplementation—such as folic acid—in migraine management remains sparse [19, 20]. Although a study comparing overall blood methylation between healthy controls and medication overuse headache (MOH) patients found no disparities [21], a significantly higher methylation level at the CORIN and CLDN9 genes in NK cells and the CCKBR gene in neutrophils was reported in another study [22]. These studies not only emphasized the importance of cell-specific methylation levels, but also indicated that we should be more careful in interpreting the results from these studies.

To move from mere correlation to causal inference, research integrating Mendelian randomization, colocalization, and mQTL data found *CFDP1*, *ICA1L*, and *SERPING1* to be core causal genes that might be affected by methylation status. Additionally, they also noticed subtype-specific methylation signatures (*RSPO2*, *CNRIP1*, and *CACNA1A* for migraine with aura; *MAPT* for migraine without aura), highlighting the value of epigenetics in clinical stratification [23]. Some study also provides additional evidence of the dynamic nature that leads to clinical presentations. An in vivo rat study showed that 43 differentially methylated regions (DMRs) had methylation changes within one hour of cortical spreading depolarization (CSD) induction. Importantly, if clinical prophylactic agents such as valproic acid (VPA) and topiramate (TPM) were given in advance, the methylation profile can be altered, which include the mechanosensitive pathways like *Piezo2* [24]. 

Besides patho-mechanism and subtype categorization, DNA methylation also serves as a promising biomarker for longitudinal monitoring and therapeutic outcome prediction. The dynamics of DNA methylation is demonstrated in a longitudinal EWAS study on chronic migraine patients, which showed that treatment response is associated with reduced methylation within HDAC4, while monthly headache days are positively correlated with the methylation status of MARK3 [25]. Additionally, a small observational study revealed that utilizing the methylation status of 9 genes participating in metabolic and neuropsychiatric pathways can predict treatment effectiveness [26]. 

More recently, novel epigenetic metrics have provided deeper insights into disease progression and treatment resistance. In 2026, two articles led by an Italian research team introduced the methylation level among migraine patients to calculate “epigenetic aging” and “epigenetic entropy”. The researchers showed that medication overuse headache patients who are resistant to treatments presented accelerated methylation aging, which was comparable with a previous study focusing on chronic pain [27, 28]. However, when compared to the control, the researchers showed the “paradoxical” age deceleration among medication overuse headache, which was postulated to be due to the anti-inflammatory effect of the painkillers [27]. The paradoxical epigenetic age deceleration observed in patients with medication overuse headache compared to healthy controls may seem counterintuitive. However, this might reflect the overall consequences of frequent anti-inflammatory use rather than the true physiological preservation. Consequently, epigenetic age alone cannot be used as reliable surrogates for clinical health status without calculating the pharmacological effect. Conversely, the accelerated epigenetic aging identified in treatment non-responders highlights its potential utility as a biomarker of refractoriness, which may be used to guide the clinicians to initiate a higher potent therapeutic strategies—such as CGRP-targeted agents—as early as possible. The other research further noted that there’s a higher epimutation burden by estimating the stochastic epigenetic mutations (SEMs) among migraine patients than in controls [29]. Moreover, the alteration rate of the SEMs, either decreasing or increasing, was the highest among those with medication overuse headache, showing a high epigenetic instability in the more severe disease phenotype [29]. 

## Non-Coding RNAs

Recently, emerging evidence has demonstrated the role of non-coding RNAs (ncRNAs) in migraine, and these findings have increased our understanding of different phenotypes as well as therapeutic approaches to migraine.

Extensive research on microRNA (miRNA) has shown its critical role in CGRP signaling, neuroinflammation, and central sensitization. For example, the hypermethylation of *miR-30a* promoter among migraine patients may drive the overexpression of *CALCA*, among migraine patients [30]. Beyond CGRP regulation, the *SIRT1* pathway and its epigenetic modulation were also frequently recognized. In a nitroglycerin-induced mouse model simulating chronic migraine, the upregulated miR-155-5p in the trigeminal nucleus caudalis (TNC) suppressed SIRT1 expression, which facilitates central sensitization [31]. This finding is consistent with the effect that miR-34a-5p can result in suppressing the expression of SIRT1, which in turn modulates the pain pathway [32]. Conversely, the downregulation of miR-382-5p and miR-22-3p was found to be associated with exacerbated pain-related behavior in another nitroglycerin-induced animal model, showing the modulating role of ncRNAs on pain sensitization [33, 34]. In comparison, the upregulated miR-653-3p after exposure to nitrogen dioxide may lead to an upregulated TRPV1, which further induced migraine-like pain responses [35]. This evidence collectively shows that the regulation of miRNA in migraine can be derived from endogenous and exogenous triggers. Besides miRNA, other ncRNAs have also emerged in the pathophysiology of migraine. For example, the participation of the circular RNA pathway driving central sensitization in migraine was recently published. In an NTG-induced mouse model, mmu_circ_0012878 is upregulated. Mechanistically, mmu_circ_0012878 can “sponge” miR-99b-5p, thereby activating the PI3K/AKT pathway, which is critical in the inflammatory process [36]. In a photophobia mouse model, the upregulated ceRNA, *NEAT1*, negatively regulated the expression of *miR-196a-5p*, which in turn increased the expression level of *Trpm3* in the trigeminal ganglion [35]. These results then exacerbate photophobic behavior in mice [35]. From these perspectives, these targets serve as important nodes in developing potential therapeutic targets for migraine.

Beyond understanding the underlying mechanism, non-coding RNAs can also serve as diagnostic biomarkers and be used to discriminate clinical subtypes. A case-control study has shown that by combining the findings of upregulated miR-27b and the downregulated miR-181a, let-7b, and miR-22, the diagnostic accuracy for migraine can reach 95.6% [37]. Similarly, the significantly upregulated miR-145-5p and miR-26a-5p, as well as the altered expression levels of miR-532-3p, miR-342-3p, and miR-758-5p in migraine patients compared to healthy controls, can also be utilized in diagnostic approaches [38, 39]. In a study focusing on the serum signature of the interictal period in migraine patients, miR-382-5p was considered a robust biomarker in distinguishing patients from healthy controls [40]. Moving from a single marker, a Taiwanese research group has taken advantage of the multi-miRNA signatures to construct a “composite genetic risk score” [41]. In this model, three miRNA signatures (miR-183, miR-25 and miR-320) effectively separated migraine patients from the control group, and by using the other four markers (miR-1307-5p, miR-6810-5p, let-7e and miR-140-3p), the disease state could be established [41]. The importance of the development of this composite genetic risk score utilizing miRNA rather than the GWAS-derived polygenic risk score should be emphasized. Because migraine is a dynamic disorder, GWAS-alone risk models derived from static germline variants can only capture baseline constitutional vulnerability rather than real-time disease fluctuations. In contrast, circulating miRNAs respond dynamically to cellular and physiologic changes, offering a more sensitive reflection of disease activity. Furthermore, the clinical transitions, such as the progression from episodic to chronic migraine or the development of medication overuse, occur along a continuous spectrum rather than as dichotomous states. Because conventional GWAS and polygenic risk scores (PRS) remain unchanged over a patient’s lifespan, they cannot capture these phenotypic shifts. Circulating miRNAs and other epigenetic marks, however, serve as biological transducers of environmental triggers as well as psychosocial stress, enabling the correlation of clinical status with neurovascular stress. Consequently, while germline genetics cannot be used for longitudinal disease monitoring due to its static nature, circulating miRNAs overcome this limitation by continuously tracking the evolving pathophysiology of migraine. Another study demonstrated the value of utilizing mitogen-activated protein kinase 14 (MAPK14) and the other four related long non-coding RNAs (lncRNAs) in distinguishing patients and healthy controls, and the predictive rate of long non-coding RNA activated by DNA damage (NORAD) was the highest at around 94.3% [42]. 

The dynamic changes of these ncRNAs provide insights into the fluctuating nature of headache episodes, i.e., the ictal and interictal phases. For instance, the significantly upregulated circulating levels of miR-34a-5p and miR-382-5p and the downregulated levels of miR-19a-3p were found during the ictal phase [38, 40]. Of note, the genes that miR-382-5p targets were related to IL10RA and GABRA5, which emphasizes the important role of inflammatory receptors and the inhibitory pathways in the formation of migraine [38]. A research studying the patient-level dynamics also demonstrated that markers participating in inflammatory processes, such as hsa-miR-3202 and other 24 miRNAs altered between ictal and interictal phases [43]. 

Clinically, though the presence of aura is canonical in migraine patients, comparing the signatures between those possessing aura and those who did not remains a clinically interesting topic. In a case-control study, the serological profiles first revealed four long non-coding RNAs (lncRNAs) that were upregulated in migraine patients, and among them, three signatures (PVT1, MEG3, LINC-ROR) differed significantly between migraine with aura and those without [44]. Another study targeting the NFkB pathway showed that the expression level of the lncRNAs of ADINR was lower in patients with aura compared with those without, while DICER1-AS1 was higher in those presenting with aura [45]. 

Beyond aura, the value of miRNA has also been investigated in the context of comparison between episodic and chronic subtypes. Few studies have collectively shown that patients diagnosed with chronic migraine with medication overuse headache (CM-MO) presented a higher level of miR-155 [46], miR-382-5p and miR-34a-5p [47] when compared with those diagnosed with episodic migraine (EM). Moreover, for CM-MO patients, the declining level of miR-382-5p represented a greater response to detoxification [47]. A small study encompassing three subjects also revealed the diverse expression of different signatures among CM patients, indicating that heterogeneity still exists [48]. Although the distinction between EM and CM remained artificial as defined by the current version of the classification, the epigenetic profiles may provide a more robust indicator in separating these patients in the future.

As for the monitoring of treatment response, an open-label study aimed to compare the change in microRNA in peripheral blood mononuclear cells (PBMCs) before and after 12 weeks of erenumab treatment in CM patients. They demonstrated that the levels of miR-34a-5p and miR-382-5p declined after the treatment [49]. The response of erenumab on EM was seen in another study, showing a declining level of hsa-miR-143-3p after the treatment [50]. Other than monoclonal antibodies, alternative management such as acupuncture has gained awareness. An animal study utilizing electroacupuncture applied over “Waiguan” and “Fengchi” on rats demonstrated altered expression of miR-34a-5p and SIRT1, a pain-modulating pathway [51]. Furthermore, a bioinformatics study showed that the inhibited expression of PECAM1 by hsa-miR-550a-3-5p is associated with a decreased pain level in nitroglycerin-induced mice [52]. 

When speaking of migraine, a similarly important issue is to manage its comorbidities. Some studies have revealed shared miRNAs signals among epilepsy and migraine, implicating a potential common pathway in these two disease entities [53]. Moreover, a study also revealed 11 known migraine miRNAs to be involved in the mechanism of major depression, which emphasizes strong clinical similarities between these two diseases [54]. In a Taiwanese study showing the elevated miR-155 and miR-126 in migraine patients were correlated with syncope frequency might imply a co-pathology with a dysregulated autonomic function and migraine [55]. 

Besides point mutations, the expression level of miRNAs related to copy number variation (CNV) also altered genetic expression. A study showed that migraine patients have a lower CNV on the 15q13.3 genetic hotspot over the gene CHRNA7, which was positively modulated with the expression level of hsa-miR-3158-5p [56]. While this review focuses on genomic DNA regulation, RNA epitranscriptomics represents another promising frontier in migraine pathophysiology. Notably, altered N6-methyladenosine (m6A) modifications have been shown to promote the secretion of CGRP and pro-inflammatory substances [57]. 

## Histone Modifications

Histone modifications, such as acetylation, dynamically alter the topological architecture between histones and DNA and lead to altered genetic expression levels. While the clinical study remained sparse, most of the findings were derived from fundamental animal studies. Following the induction of cortical spreading depression (CSD), there’re decreased H3K4 methylation and increased methylation over H3K9, which both lead in a direction toward lesser transcription of the genes [58]. Though some studies mentioned histone deacetylase (HDAC), specifically HDAC6, as a potential mechanism in migraine [59] the substrates of HDAC6 were on microtubules rather than the chromatin and therefore are out of the scope of this review. Nevertheless, the endogenous inhibitory effect of valproic acid was postulated to participate in the epigenetic modification pathways, exerting its preventive mechanism in migraine [60]. 

**Table 1:** Common epigenetic modifications and their effects on migraine

Epigenetic modifications	Mechanism	Examples and plausible implications
DNA methylation	DNA methylation at specific loci regulates the level of gene expression across distinct tissues [62].	The regulation of CGRP-pathway-related proteins, i.e., CALCA and RAMP1, were regulated by their methylation levels [11, 12]. Therefore, future therapeutic approaches may focus on alternating the methylation status of these critical proteins.
Non-coding RNAs (ncRNAs)	Though not participated in encoding functional proteins, ncRNAs can played an important role in modifying the post-transcriptional expression levels of certain genes [63].	miRNA signatures found to present distinctly between disease and control groups as well as different disease states was utilized to construct effective models in diagnosing approaches. [41].
Histone modifications	The modifications, especially the acetylation, of different histone proteins altered the expression level of the overlapping regions [64].	The histone modification in a rat model was seen after the induction of CSD, indicating this mechanism in the presentation of migraine [58]. The endogenous effect of deacetylation from valproic acid may partially explain its clinical effect [60].

## Discussions

The advance in novel techniques has broadened our understanding of the genetic and epigenetic landscape of migraine. In this review, we synthesized current evidence regarding the epigenetic profiles of migraine, especially DNA methylation, non-coding RNAs, and histone modification. (Table 1) These results have gained further insights into migraine pathophysiology, the chronification of migraine from episodic to chronic presentations, the potential differences between migraine with and without aura, as well as emerging treatment strategies focusing on these epigenetic pathways [61]. 

Despite promising, there still remain several limitations warranting consideration within this review. First, the restriction of the research method of this study may not cover all the existing results in other databases. Second, many of the results are derived from animal models, and the translation to clinical use remains a large gap. Third, the epigenetic markers detected in peripheral tissues in many studies may not truly reflect intracranial pathology, not to mention the specificity of trigemino-vascular tissues. Therefore, further studies focusing on tissue-specific research, the interaction with the environment, as well as translational investigations are still required to validate these findings.

## Conclusions

The development and the endophenotype of migraine can be modulated epigenetically. Further studies are required to validate these results.

## Key References

- Sutherland HG, Jenkins B, Griffiths LR. Genetics of migraine: complexity, implications, and potential clinical applications. Lancet Neurol. 2024;23(4):429-46. doi: 10.1016/S1474-4422(24)00026-7.
  - ○ This article provides a detailed investigation into how migraines relate to associated biomarkers and comorbidities.
- Ihara K, Casillo F, Dahshan A, Genc H, Jusupova A, Karbozova K, et al. Are we closer to achieving precision medicine for migraine treatment? A narrative review. Cephalalgia. 2024;44(9):3331024241281518. doi: 10.1177/03331024241281518.
  - ○ This review examines the clinical, biological profiles that serve as signatures for monitoring migraine treatment responses.
- Tao Y, Cai X, Sun Y, Lu W, Wang S, Huang Z, et al. The emerging role of epigenetic regulation in pain sensitization associated with headache disorders. J Headache Pain. 2025;27(1):15. doi: 10.1186/s10194-025-02244-4.
  - ○ This review provides the information of how the epigenetic mechanisms participates in the pathophysiology of headache disorders.

## Data Availability

No datasets were generated or analysed during the current study.
